# Effectiveness and safety of fully oral modified shorter treatment regimen for multidrug-resistant tuberculosis in Georgia, 2019–2020

**DOI:** 10.4081/monaldi.2021.1679

**Published:** 2021-01-14

**Authors:** Teona Avaliani, Yuliia Sereda, Hayk Davtyan, Nestani Tukvadze, Tamar Togonidze, Nana Kiria, Olga Denisiuk, Ogtay Gozalov, Sevim Ahmedov, Arax Hovhannesyan

**Affiliations:** 1 National Center for Tuberculosis and Lung Diseases, Tbilisi, Georgia; 2 World Health Organization, Regional Office for Europe, Copenhagen, Denmark; 3 Tuberculosis Research and Prevention Center, Yerevan, Armenia; 4 Alliance for Public Health, Kyiv, Ukraine; 5 United States Agency for International Development, Washington DC, USA

**Keywords:** MDR-TB, modified all-oral shorter treatment regimen, rifampicin resistance, time to culture conversion, adverse drug safety monitoring

## Abstract

Tuberculosis treatment is challenging, especially among people with drug-resistant forms of tuberculosis. The introduction of fully oral modified short treatment regimen has a great potential to shorten duration of treatment, improve safety and ultimately increase treatment success rate. In 2019 Georgia has piloted the modified fully oral shorter treatment regimen in a routine programmatic condition. Our study aimed to evaluate effectiveness and safety of the modified shorter treatment regimen in Georgia among the first 25 consecutively enrolled patients with rifampicin-resistant tuberculosis with proven sensitivity to fluoroquinolone and without prior exposure to second-line tuberculosis drugs. Regimen consisted of 9-month daily administration of bedaquilline, linezolid, levofloxacin, clofazimine and cycloserine. Study patients were enrolled between March-August 2019. We used a national electronic surveillance system, medical records and active TB drug-safety monitoring and management database to extract study related data. The mean age of the study participants was 48 years, 68% were male, 8% were HIV positive, 16% had diabetes and 12% tested positive for hepatitis C infection. The median time to culture conversion among 16 patients who were culture positive at treatment initiation was 1.0 (95% CI: 1.0–2.0) month. Of those, by the end of treatment 15 patients converted to negative. Out of 25 patients in the study cohort 22 (88%) had successful treatment outcome, one patient (4%) died and two (8%) were lost to follow up. The regimen was largely well tolerated. Three patients (12%) experienced serious adverse events - two cases were possibly related to TB drugs in the regimen. Seven patients developed adverse events of interest in eight instances, including musculoskeletal (twice), psychiatric, gas-trointestinal disorders, hepatotoxicity, peripheral neuropathy, cardiotoxicity and myelosuppression (once each). In four patients (16%) the duration of the treatment was extended beyond nine months due to insufficient radiological improvements. Our findings demonstrate that good treatment outcomes are achievable in people with fluoroquinolone-sensitive tuberculosis within routine programmatic conditions using fully oral modified short treatment regimen. The extensive use of fully oral modified shorter treatment regimen in Georgia and other high priority countries in the World Health Organization European Region is warranted.

## Introduction

Despite the availability of effective and affordable treatment discovered 70 years ago, control of tuberculosis (TB) around the world remains cumbersome. Annually, about 10 million people fell ill with TB and only seven million are identified by health systems [1]. TB control is challenged by the emergence of drug resistance (DR-TB), defined as the disease caused by a bacterial strain that is resistant to at least rifampicin, one of the most effective medications to treat TB. In the case of the multidrug resistant TB (MDR-TB) the bacterial strain is resistant to at least both rifampicin and isoniazid. Globally RR/MDR-TB is reported among 3.4 % of new cases and 18 % of those with previous history of TB treatment [1].

TB control presents a unique challenge especially for the World Health Organization (WHO) European region; accounting for only 3% of global tuberculosis burden, about 24% of drug-resistant tuberculosis cases occur in the WHO European region [1]. Despite of accelerated decline in overall tuberculosis notification rates observed in recent years in the Region, per capita rate of drug-resistant tuberculosis is increasing. If this trend is sustained, there is a risk that the observed decline in the tuberculosis burden will be halted [2].

Georgia is one of the 18 high priority countries for TB in the WHO European Region [3]. In 2018 the incidence rate of TB cases per 100,000 in Georgia was 52, RR/TB was diagnosed in 12% of new and 30% of previously treated cases [4].

Treatment of DR-TB remains a difficult task due to its long duration, adverse events and costs. RR/MDR-TB treatment outcomes are usually poor: among RR/MDR-TB patients enrolled into treatment in 2016 in WHO European region only 57.6% were successfully treated [5].

Until recently, the only treatment option for people with RR/MDR-TB was up to 20-month duration regimen with different combination of second-line drugs, which was toxic, expensive, unacceptably long and often was perceived as more challenging than the disease itself [6]. Besides its poor tolerability, these regimens commonly were characterized by poor adherence to treatment and led to treatment discontinuation and unfavourable treatment outcomes [7–9]. Georgia was one of the first countries in the WHO European Region to introduce novel and repurposed drugs as a part of compassionate use. The effectiveness and safety of treatment of patients who received new and repurposed drugs in pilot projects in Georgia were encouraging [10,11]. In 2019, Georgia introduced fully oral shorter modified treatment regimens (mSTR) before WHO would release key changes in DR-TB management guidelines in February 2020, including operational guidance on mSTR [12]. For the first time in decades a new regimen with new TB drugs was introduced to treat people with RR/MDR-TB, offering numerous potential advantages. The shorter duration of the treatment, the replacement of an injectable agent with a medication administered orally, and the use of drugs with better safety profiles, were the key improvements of this regimen to increase the likelihood of successful treatment for the people with DR-TB.

Our study aimed to evaluate the effectiveness and safety of the modified shorter RR/MDR-TB regimen in Georgia. More specifically, our study was designed to assess interim and end of treatment outcomes among patients enrolled into mSTR and describe the spectrum of Serious Adverse Events (SAE), Adverse Events of Interest (AEI) of grade 3 and 4, and their management practice.

## Materials and Methods

### Study design

This was a descriptive study involving analysis of routine programmatic data collected prospectively.

### Study population

All laboratory confirmed RR/MDR-TB patients who have started treatment with modified fully oral shorter RR/MDR-TB regimen within the NTP from March till August 2019 were included in the study.

### Diagnosis of RR/MDR-TB

Passive case-finding is the main method of TB detection in Georgia. Primary health care providers are responsible for identifying presumptive TB cases and referring them to specialized TB service units for diagnosis. Diagnosis of TB is established by direct sputum smear microscopy and GeneXpert MTB/RIF, supported by chest radiography and culture. According to routine surveillance data, in 2019 about 85% of notified new and relapse TB patients were diagnosed using WHO recommended rapid tests. The National Reference Laboratory in Tbilisi performs the full range of TB laboratory investigations, including first and second-line Line-Probe Assay, culture testing on liquid and solid media and phenotypic Drug Susceptibility Tests (DST) for first and second-line drugs. External quality control of DST is implemented annually by Supranational Reference Laboratory of Tropical Institute of Medicine in Antwerp, within NRL-SRL partnership.

### Treatment of RR/MDR-TB

Decision of enrolment into DR-TB treatment, regimen choice and further modifications are undertaken by the national DR-TB consilium, which is a multidisciplinary team consisting of TB-doctors, laboratory, surveillance and adherence support specialists. Typically, patients who start second-line treatment are hospitalized during the first 2 months of the treatment. Then the treatment is continued under direct observation of outpatient health facility near to the area of residence.

People with RR/MDR-TB are eligible for mSTR regimen if they are affected by a *Mycobacterium tuberculosis* strain that is sensitive to fluroquinolones and had not been previously treated for more than one month with second-line drugs, and they do not have extensive forms of disease [13]. The standard mSTR regimen includes 9-month daily administration of bedaquiline, linezolid, levofloxacin, clofazimine and cycloserine. The dosage of drugs included in regimen are shown in Supplementary Table 1. Monitoring of the treatment is implemented according to the national guidelines, which outline frequency, timing and types of clinical and laboratory tests and examinations to ensure timely identification of adverse events. More specifically, treatment progress is monitored by sputum smear microscopy and culture testing on liquid media. The monthly clinical evaluation includes assessment of peripheral neuropathy, checking colour vision, visual acuity, testing blood for electrolytes, creatinine, full blood count and liver function. Electrocardiography (ECG) is performed once every two weeks in the first month and then once a month. Chest radiography is performed every three months. All health care providers involved in management of DR-TB were trained on new treatment guidelines and in identification, recording, reporting and management of adverse events (AEs).

### Recording

The NTP in Georgia maintains real-time case-based surveillance system for all TB patients, which includes key demographic and clinical data. In addition, NTP maintains system of active drug-safety monitoring and management in line with international recommendations. All SAEs and AEIs are captured in the standard AEs reporting paper forms and transferred to the department of pharmacovigilance, where those data are entered in the electronic system.

### Definitions

Sputum conversion is defined as two consecutive negative cultures from samples collected at least 30 days apart. Time to culture conversion is the interval between the date of treatment initiation and the date of collection of the first of two negative cultures. End of treatment outcomes definitions were the following: “cured” - treatment completed without evidence of failure AND three or more consecutive cultures taken at least 30 days apart are negative at the end of treatment; “treatment completed” - treatment completed without evidence of failure BUT no record that three or more consecutive cultures taken at least 30 days apart are negative at the end of treatment; “treatment failed” - treatment terminated or need for permanent regimen change of ≥2 anti-TB drugs because of lack of conversion, bacteriological reversion after conversion to negative, evidence of acquired resistance to drugs in the shorter regimen or adverse drug reactions leading to the change of at least two anti-TB drugs in the regimen; “died” – a patient who dies for any reason during the course of treatment; “lost to follow-up” – a patient whose treatment was interrupted for 2 consecutive months or more. Favourable outcome is defined as a combination of cured and treatment completed. Unfavourable outcome is the combination of loss to follow-up, death and failure.

SAE is any untoward medical occurrence irrespective of the cause that resulted in death, was life-threatening, required hospitalization, resulted in significant disability or was considered otherwise medically important. Adverse event of interest is an adverse event documented to have occurred during clinical monitoring.

### Data source

Clinical and demographic data were extracted from routine electronic case-based surveillance system. Data on adverse events were extracted from the aDSM database, as well as from individual treatment charts. Two databases were merged using patient ID as a key variable to match the observations. Additional data extracted from medical charts were entered manually.

### Variables

Variables for each of the study objectives were chosen from the list of data variables available in the NTP database and standardized SAE reporting forms. Those included key demographic and clinical characteristics, such as age, sex, co-morbidities, treatment outcome, dates of start of treatment, treatment outcome, culture test results, date of culture test results, AE, dates and characteristics of AEs, *etc.*

### Statistical analysis

We described socio-demographic and clinical characteristics of the study participants using frequencies and percentage, and continuous data were summarized using means and standard deviations if data were normally distributed and medians and interquartile ranges if they were skewed. We summarized interim treatment outcome by calculating monthly cumulative proportions of patients with culture conversion during the first six months of treatment. We calculated culture conversion among patients with positive culture result at the date of treatment initiation. Time to culture conversion was measured using a cumulative incidence function to account for competing risks of death and loss to follow up. Among patients with evaluated treatment outcome, we calculated proportions of patients with treatment success (cured or treatment completed) and unfavourable treatment outcome (failure, death or loss to follow up). Serious adverse events and adverse events of interest were summarized by type of event, severity, seriousness, time to onset, duration, management, and their outcome. Regarding non-resolved AE, duration was calculated as a time interval between the event onset and treatment outcome. Incidence rate of adverse events was measured as a total number of events over the overall follow-up time, from treatment initiation to treatment outcome. Analysis was done using R, version 3.5.2 software (^©^R Foundation for Statistical Computing, 2016).

## Results

Of 25 patients included in the study, 17 (68%) were male. The average [standard deviation (SD)] age of the study participants was 48 (±16) years and ranged from 18 and 77 years. Of total two (8%) were HIV positive, four (16%) patients had diabetes and three (12%) tested positive for hepatitis C infection. At the baseline 16 patients (64%) were culture positive, 7 (28%) were negative and in two patients (8%) the culture results were contaminated (Table 1).

Of 25 patients in study population, 24 started TB treatment with Bdq-Lzd - Lfx -Cfz - Cs regimen. In one patient Lzd was not initiated due to HIV, and a high dose Isoniazid was started instead. In another patient with documented sensitivity to FQ genotypic DST at the start of the treatment the phenotypic DST results received at the second month of treatment showed additional resistance to fluoroquinolones; therefore, levofloxacin in the regimen was replaced with delamanid.

Mean (SD) treatment duration was nine 9.2 (±2.2) months ranging from 0.4 to 13.5 months. 21 patients completed the treatment within nine months while in four patients the treatment was extended beyond nine months because of insufficient radiological progress.

### Effectiveness

**Figure 1.**
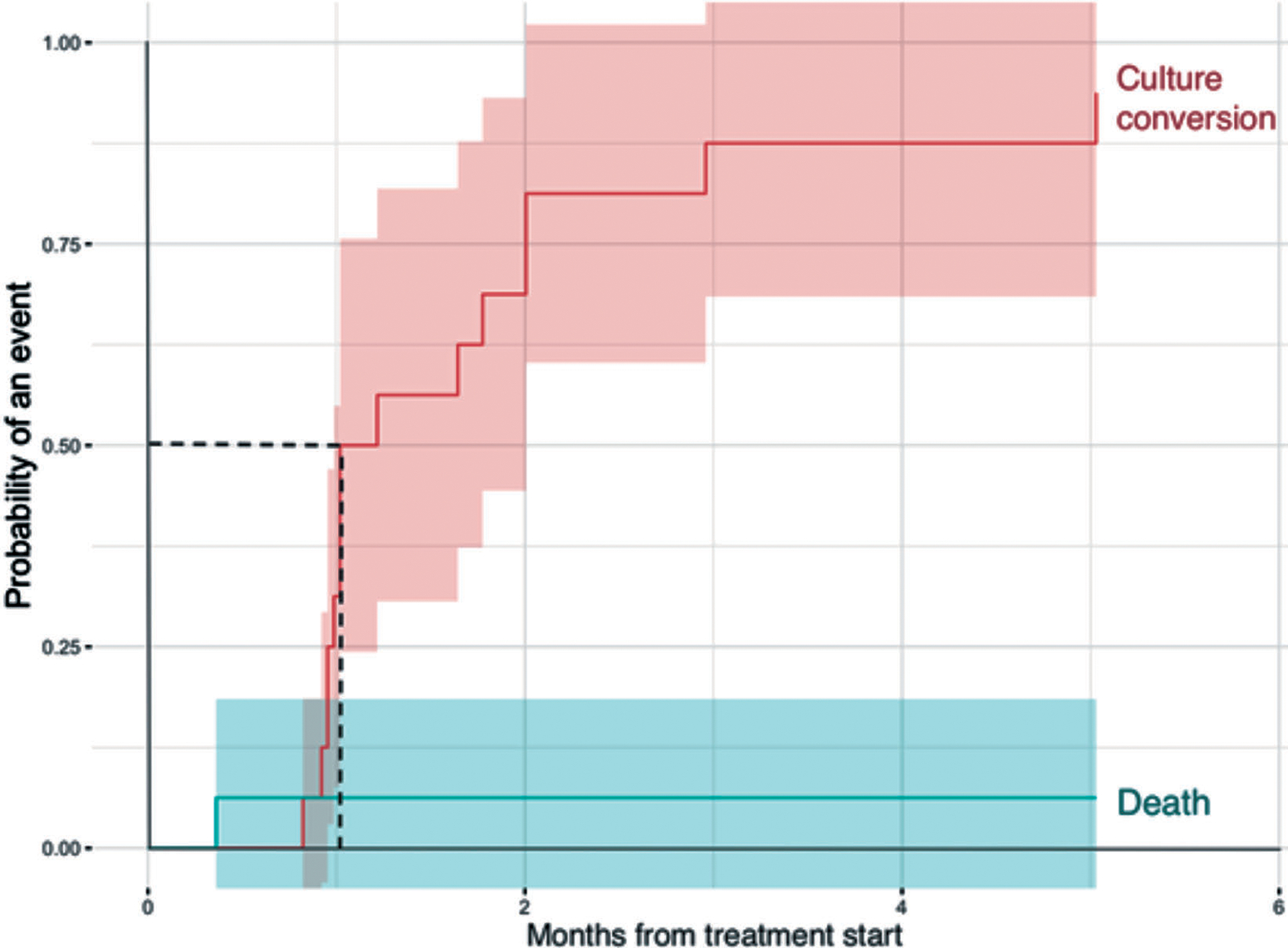
Time to culture conversion among 16 patients enrolled in treatment with modified shorter RR/MDR-TB regimen from March till August, Georgia.

At the 4^th^ month of treatment of 16 patients, who were culture positive at treatment initiation, 14 (88%) converted. At the 6th month, 15 (94%) patients had converted. Median time to culture conversion was 1.0 month [95% confidence interval (CI): 1.0–2.0]. Conversion of the last patient occurred on the 5^th^ month of the treatment. The time to culture conversion plot is presented in Figure 1. None of the patients who had culture conversion re-converted during the course of treatment. Likewise, no patients who were culture-negative at treatment initiation became culture-positive.

Out of all 25 patients in the study cohort, 22 (88%, 95% CI: 69–98%) had successful treatment outcome. One (4%, 95% CI: 0.1–20%) patient died at the first month of treatment and two (8%, 95% CI 1–26%) were lost to follow up (Table 2).

### Safety

A total of three [12%, 95% CI: 2.5–31.2%; incidence rate (IR): 1.3 per 100 person-months, 95% CI: 0.06–0.80] patients experienced three SAE, cardiotoxicity, hepatotoxicity, death, two were related to TB drugs (Table 3). Both drug-related SAE required interruption of the suspected drug and were resolved within one month. Another SAE with fatal outcome was evaluated as not related to TB drug. SAEs occurred at 0.4, 2.9 and 5.3 months.

Another eight AEIs were reported in seven (28%, 95% CI: 12–49%; IR: 3.5 per 100 person-months, 95% CI: 0.32–1.44) patients. Of all eight AEIs, musculoskeletal disorder appeared in two instances, while cardiotoxicity, gastro-intestinal disorder, hepatotoxicity, myelosuppression, psychiatric disorder and peripheral neuropathy appeared once each.

Of 8 AEI, three were registered during the first month of treatment, one between the second and third month, two between the fifth and sixth months, and one between the seventh and eighth month.

For managing of SAE/AEI in one instance the suspected drug was permanently withdrawn, in five instances drug was interrupted, and in another instance the dose of suspected drug was reduced. In eight instances (73%) the outcome of the SAE/AEI was resolved. Two cases (18%) had unresolved adverse events at the time of discharge and no follow up data was available for them. Median time to SAE/AEI was 77 days (IQR: 20 – 162 days, range: 11 – 221 days). Median duration of SAE/AEI was 15 days (IQR: 9 – 89 days, range: 0 – 211 days).

## Discussion

In our study, we found that the 9-month-long treatment in the first sample of patients receiving all-oral modified regimen was highly effective. The culture conversion was achieved quite fast and the regimen was largely well tolerated.

The median time to conversion was around one month, which is very short compared with the standard treatment showing 64 days (IQR 58 – 106 days) median time to conversion in a study conducted in Georgia among RR/MDR-TB patients enrolled into treatment between 2009 to 2012. Moreover, in our study most of initially culture-positive participants (94%, 15/16) achieved culture conversion, while with conventional treatment in above-mentioned study, only 71% of patients converted to negative by the end of treatment [14]. In other studies, conducted around the world among RR/MDR-TB patients, median time to culture conversion ranged from 61 to 196 days [14–18]. It is noteworthy that culture conversions were permanent for the entire duration of the follow-up, and none of the patients had a re-conversion during the course of treatment. Since culture conversion is used as an early biomarker in treatment outcomes, the faster conversion is encouraging and indicates that provided regimen is effective.

The high rate of conversion could be attributed to the fast-sterilizing effect of novel drugs in the regimen, as early conversion is consistently reported across cohorts using novel medicines [19–21]. For instance, in a cohort of RR/MDR-TB patients on bedaquiline-containing short regimen in Belarus, 87% achieved a culture conversion at the 6^th^ month of the treatment [22].

The observed 88% treatment success rate in our study population compares favourably with national average of 65.5% treatment success reported in 2016 RR/MDR-TB treatment cohort. Our findings are comparable with the 73% treatment success rate of the 987 RR/MDR-TB patients enrolled in all-oral bedaquiline-containing short treatment regimen in South Africa [23]. Available data on treatment effectiveness among patients enrolled into treatment using novel drugs in our region demonstrate similar promising results. Thus, study from Belarus achieved 92% treatment success rate among RR/MDR-TB patients enrolled in bedaquiline-containing treatment [22]. Only two patients (8%) interrupted the treatment. This is about two times lower in comparison with 2016 RR/MDR-TB cohort showing 19% lost to follow up rate supporting the evidence that short duration of the treatment reduces the likelihood of treatment interruption compared to the longer regimens [24].

We believe that such high treatment success rate was attributable to short duration, simplicity of administration (without daily painful injections) and convenience of the fully oral mSTR. However, we also acknowledge that low drug resistance profile and prior non-exposure to second-line drugs, which are selection criteria for mSTR regimen, also notably contributed to the observed high effectiveness of the treatment.

In our treatment cohort, only three people with TB experienced SAEs (12%, 95% CI: 2.5%−31.2%), which is reasonably low, although the uncertainty is very large due to small number of observations. Our finding is consistent with the global sDSM project report comprising 26 countries, which showed 11% of SAE in RR/MDR cohort treated with regimens containing novel and repurposed drugs [19]. However, we also acknowledge that the global project includes patients with extensive drug resistance, and regimens which contains second line injectables. A meta-analysis of studies focusing on short course MDR regimens identified that 18% of patients receiving these regimens experienced a grade 3 or grade 4 adverse events [25].

To our knowledge this is one of the first studies around the world reporting data on the effectiveness and safety of the fully oral modified short treatment regimen under routine programmatic conditions. The patients enrolled into the treatment were directly observed over the entire study duration, all laboratory tests and examinations for the monitoring of the treatment and AE were conducted in-line with national protocols, which allowed to produce reliable and accurate data. For the treatment monitoring the culture testing was done on liquid media, which is known to be more sensitive compared to solid media [26], indicating on reliability of the data produced by the study.

A major limitation is the small sample of patients, which precluded inferential analysis. Thus, we reported only descriptive statistics. Seven patients at the start of treatment had negative or contaminated culture, which further reduced the number of observations for the interim effectiveness analysis. Another limitation of this study is that the participants were recruited in a single country, therefore, the result cannot be generalizable. Even though 12-months follow-up outcome data are not yet available for our cohort, sharing the end-of treatment outcome data have paramount importance for the countries of our Region, most of which will start the introduction of mSTR regimen under routine programmatic conditions with technical guidance of WHO European Region.

Despite of small sample size and lack of post-treatment follow-up data, our findings demonstrate that excellent treatment outcomes are achievable in people with fluoroquinolone-sensitive RR/MDR-TB within routine programmatic conditions using mSTR regimen and this, thereby, underscore an urgent need to expand the use of mSTR regimen in Georgia and other high priority countries in the WHO European Region.

## Conclusions

The current study yields promising evidence for the use of fully oral mSTR in treatment of RR/MDR-TB patients in terms of effectiveness. Safety of these regimens should be closely monitored, while the scale up of the regimen use occurs in the country or in other locations. Observed 88% effectiveness in this cohort is much higher than it has ever been recorded previously in Georgia. If such results are confirmed in further cohorts, this regimen will provide good potential to meet ambitious goal to end TB by 2035 in Georgia.

## Supplementary Material

SUPPLEMENTARY MATERIAL

## Figures and Tables

**Table 1.** Baseline characteristics of patients enrolled in treatment with modified shorter RR/MDR-TB regimen from March till August, Georgia.

Characteristics	n	%
Total	25	100
Mean age (standard deviation), years	48	16
Age groups, years		
18–45	11	44
46–77	14	56

Gender		
Male	17	68
Female	8	32
HIV status		
Positive	2	8
Negative	23	92

Diabetes		
Yes	4	16
No	21	84
Hepatitis C		
Yes	3	12
No	22	88

Other comorbidities		
Yes	9	36
No	16	64
Baseline culture result		
Positive	16	64
Negative	7	28
Contaminated	2	8

Data is summarized as n and % unless otherwise stated.

**Table 2.** Interim and end-of treatment outcomes among patients (N=25) enrolled in treatment with modified shorter RR/MDR-TB regimen from March till August, Georgia.

	n/N	%
Interim treatment outcomes°		
Cumulative sputum culture conversion		
Month 1	5/16	31
Month 2	13/16	81
Month 3	14/16	88
Month 4	14/16	88
Month 5	15/16	94
Month 6	15/16	94
End of treatment outcomes		
Treatment success	22/25	88
Cured	18/25	72
Treatment completed	4/25	16
Unsuccessful outcome	3/25	12
Treatment failed	0/25	0
Died	1/25	4
Lost to follow-up	2/25	8

° The analysis is restricted to patients with positive culture result at the time of treatment initiation.

**Table 3.** Frequency, spectrum, severity, management and outcomes of serious adverse events and adverse events of interest among patients enrolled in treatment with modified shorter RR/MDR-TB regimen from March till August, Georgia.

Total, n (%)		Adverse event class							
			Cardiotoxicity	Cardiovascular disorder	Gastrointestinal tract	Hepatotoxicity	Musculoskeletal disorders	Myelosupression	Peripheral Neuropathy	Psychiatric issues
Total number of adverse events°		11 (100)	2	1	1	2	2	1	1	1
Proportion of patients with adverse events*		9 (36)	2	1	1	1	2	1	1	1

Adverse events of interest°		8 (73)	1	–	1	1	2	1	1	1
Serious adverse events°		3 (27)	1	1	–	1	–	–	–	–

Grade°^#^	Mild	–	–	–	–	–	–	–	–	–
	Moderate	–	–	–	–	–	–	–	–	–
	Severe	2 (18)	1	–	–	1	–	–	–	–
	Life-threatening	–	–	–	–	–	–	–	–	–
	Fatal	1 (9)	–	1	–	–	–	–	–	–
	Unknown	8 (73)	1	–	1	1	2	1	1	1
Management°	Dose maintained	3 (27)	–	–	1	1	–	–	–	1
	Dose reduced	1 (9)	–	–	–	–	–	–	1	–
	Drug interrupted	5 (45)	2	–	–	1	1	1	–	–
	Permanent withdrawal	1 (9)	–	1	–	–	1	–	–	–
	Not applicable	1 (9)	–	1	–	–	–	–	–	–

Outcome°	Resolved	8 (73)	2	–	1	1	1	1	1	1
	Not resolved	2 (18)	–	–	–	1	1	–	–	–
	Fatal	1 (9)	–	1	–	–	–	–	–	–

° Denominator for the percentage is the total number of adverse events (n=11)

* denominator for the percentage is the total number of patients (n=25)

# grading was recorded only for serious adverse events.
