# Supplementary material for: Effectiveness and safety of fully oral modified shorter treatment regimen for multidrug-resistant tuberculosis in Georgia, 2019–2020
Source: Monaldi Arch Chest Dis. Author manuscript; Available in PMC 2022 Aug 20. (PMC9391985; doi:10.4081/monaldi.2021.1679)
Supplement: SUPPLEMENTARY MATERIAL [file NIHMS1827070-supplement-SUPPLEMENTARY_MATERIAL.pdf]

SUPPLEMENTARY MATERIAL

DOI: 10.4081/monaldi.2021.1679

Effectiveness and safety of fully oral modified shorter treatment regimen for multidrug-resistant tuberculosis in Georgia, 2019-2020

Teona Avaliani[1], Yuliia Sereda[2], Hayk Davtyan[3], Nestani Tukvadze[1], Tamar Togonidze[1], Nana Kiria[1], Olga Denisiuk[4], Ogtay Gozalov[2], Sevim Ahmedov[5], Arax Hovhannesyan[2]

[1]National Center for Tuberculosis and Lung Diseases, Tbilisi, Georgia; [2]World Health Organization Regional Office for Europe, Copenhagen, Denmark; [3]Tuberculosis Research and Prevention Center, Yerevan, Armenia; [4]Alliance for Public Health, Kyiv, Ukraine; [5]United States Agency for International Development, Washington DC, USA

Correspondence: Teona Avaliani, National Center for Tuberculosis and Lung Diseases, 8 Achara str, Tbilisi 0101, Georgia. Tel. +995.593 48 89 65. E-mail: teoavaliani1@gmail.com

Key words: MDR-TB; modified all-oral shorter treatment regimen; rifampicin resistance; time to culture conversion; adverse drug safety monitoring.

Creative Commons Attribution 3.0 license

Supplementary Table 1. Dosage of drugs used for the standards modified short treatment regimen in RR/MDR-TB patients in Georgia, 2019-2020.

Supplementary Table 1. Dosage of drugs used for the standards modified short treatment regimen in RR/MDR-TB patients in Georgia, 2019-2020.

Drug	Drug doses by weight band of adult patients				
	30-35 kg	36-45 kg	46-55 kg	56-70 kg	>70 kg
Bedaquiline (Bdq)	400 mg for first 2 weeks; then 200 mg 3 times a week				
Linezolid (Lzd)	300 mg	300 mg	600 mg	600 mg	600 mg
Levofloxacin (Lx)	750 mg	750 mg	1000 mg	1000 mg	1000 mg
Clofazimine (Cfz)	100 mg	100 mg	100 mg	100 mg	100 mg
Cycloserine (Cs)	500 mg	500 mg	750 mg	750 mg	750 mg
Delamanid (Dlm)	200 mg	200 mg	200 mg	200 mg	200 mg

Creative Commons Attribution 3.0 IGO

pagepress
